# Defining a Methylation Signature Associated With Operational Tolerance in Kidney Transplant Recipients

**DOI:** 10.3389/fimmu.2021.709164

**Published:** 2021-08-20

**Authors:** Ramon M. Rodriguez, María P. Hernández-Fuentes, Viviana Corte-Iglesias, María Laura Saiz, Juan José Lozano, Ana R. Cortazar, Isabel Mendizabal, María Luisa Suarez-Fernandez, Eliecer Coto, Antonio López-Vázquez, Carmen Díaz-Corte, Ana M. Aransay, Carlos López-Larrea, Beatriz Suarez-Álvarez

**Affiliations:** ^1^Translation Immunology Laboratory, Instituto de Investigación Sanitaria del Principado de Asturias-ISPA, Oviedo, Spain; ^2^Red de Investigación Renal (REDinREN), Instituto de Salud Carlos III (ISCIII), Madrid, Spain; ^3^Lipids in Human Pathology, Institut d’Investigació Sanitària Illes Balears (IdISBa, Health Research Institute of the Balearic Islands), Palma, Spain; ^4^MRC Centre for Transplantation, King’s Health Partners, Guy’s Hospital, King’s College London, London, United Kingdom; ^5^Bioinformatics Platform, Centro de Investigación Biomédica en Red de Enfermedades Hepáticas y Digestivas (CIBEREHD), Barcelona, Spain; ^6^Genome Analysis Platform, Center for Cooperative Research in Biosciences (CIC bioGUNE), Derio, Spain; ^7^Center for Cooperative Research in Biosciences (CIC bioGUNE), Basque Research and Technology Alliance (BRTA), Derio, Spain; ^8^Ikerbasque, Basque Foundation for Science, Bilbao, Spain; ^9^Nephrology Department, Hospital Universitario Central de Asturias, Oviedo, Spain; ^10^Genética Molecular, Hospital Universitario Central Asturias, Oviedo, Spain; ^11^Immunology Department, Hospital Universitario Central de Asturias, Oviedo, Spain; ^12^Centro de Investigación Biomédica en Red de Enfermedades Hepáticas y Digestivas (CIBERehd), Instituto de Salud Carlos III (ISCIII), Madrid, Spain

**Keywords:** epigenetics, DNA methylation, operational tolerance, kidney transplant, rejection

## Abstract

Operational tolerance after kidney transplantation is defined as stable graft acceptance without the need for immunosuppression therapy. However, it is not clear which cellular and molecular pathways are driving tolerance in these patients. We performed genome-wide analysis of DNA methylation in peripheral blood mononuclear cells from kidney transplant recipients with chronic rejection and operational tolerance from the Genetic Analysis of Molecular Biomarkers of Immunological Tolerance (GAMBIT) study. Our results showed that both clinical stages diverge in 2737 genes, indicating that each one has a specific methylation signature associated with transplant outcome. We also observed that tolerance is associated with demethylation in genes involved in immune function, including B and T cell activation and Th17 differentiation, while in chronic rejection it is associated with intracellular signaling and ubiquitination pathways. Using co-expression network analysis, we selected 12 genomic regions that are specifically hypomethylated or hypermethylated in tolerant patients. Analysis of these genes in transplanted patients with low dose of steroids showed that these have a similar methylation signature to that of tolerant recipients. Overall, these results demonstrate that methylation analysis can mirror the immune status associated with transplant outcome and provides a starting point for understanding the epigenetic mechanisms associated with tolerance.

## Introduction

The Kidney transplantation is the most suitable treatment for end-stage renal disease. Unfortunately, most of these patients require long-term immunosuppression (IS), which is associated with a higher risk of infection, malignancies and metabolic diseases that ultimately reduce graft survival (1, 2). A small fraction of these patients spontaneously develop operational tolerance, i.e., stable graft acceptance without IS and with apparently normal immune competence (3). In general, kidney transplant recipients (KTR) with operational tolerance have been identified accidentally through noncompliance, post-transplant development of cancer, or due to pregnancy. The estimated prevalence of spontaneous operational tolerance is very low. Moreover, there is no efficient tolerance-inducing protocol, because of the high risk of rejection associated with IS withdrawal and the difficulty of predicting transplantation outcome. In this context, it is essential to develop reliable non-invasive biomarkers to identify potentially tolerant patients and to minimize IS drugs. With this aim, gene expression studies using microarray technology have generated transcriptional signatures associated with operational tolerance (4–6). Nonetheless, the omic field has undergone great advances that have allowed much more complex data to be generated that cover not only gene transcription but also the epigenetic mechanisms that drive these transcriptional programs. In recent years, epigenetic mechanisms, such as DNA methylation and histone marking, have been shown to be essential during the differentiation and activation of immune cells (7–10). Consequently, identifying the epigenetic networks in peripheral blood of KTR could yield new insights into the immune mechanisms associated with operational tolerance.

In this study, we set out to analyze DNA methylation patterns in KTR with chronic rejection and operational tolerance from the Genetic Analysis of Molecular Biomarkers of Immunological Tolerance (GAMBIT) study. By using high-density microarray technology, we were able to analyze DNA methylation in 850,000 genomic regions. Our results indicate that DNA methylation changes are associated with transplant outcome, and that operational tolerance is associated with the acquisition of different methylation profiles in genes related to B and T cell signatures, which could condition the immune response mediated by these cell types.

## Materials and Methods

### Patients and Samples

Blood samples were acquired from KTR recruited as part of the Genetic Analysis & Monitoring of Biomarker of Immunological Tolerance study (GAMBIT, Research Ethics Reference 09/H0713/12, UK) and healthy donors (HC; n = 7) from the Asturias Transfusion Centre, Spain. All individuals gave their written informed consent in accordance with the Declaration of Helsinki. The study included KTR from different clinical groups: tolerant (TOL; n = 9), chronic rejection (CR; n = 6), clinically stable patients with only low doses of prednisone (MO; n = 7) or on standard triple therapy (TT; n = 7). Tolerant patients were defined as having a functionally stable transplanted kidney without IS for more than 1 year and serum creatinine (SCr) levels less than 10% rise in the last twelve months. Chronic rejection was defined as patients with graft dysfunction despite adequate IS, and proved by a recent biopsy showing signs of immunologically rejection (TCMR, ABMR or mixed) in according to BANFF criteria. KTR were considered stable when its SCr levels were lower than 1.8 mg/dl and less than 10% rise in the last twelve months. In MO group, the withdrawal of immunosuppression, except prednisone, was conducted due to clinical reasons and took place more than 1 year before take sample. In TT group, all KTR were treated with prednisolone, calcineurin inhibitor (CNI, cyclosporine or tacrolimus) and the anti-proliferative agent mycophenolate-mofetil. HC donors were age- and sex-matched to transplanted patients. Patient characteristics and immunosuppressant regimens are shown in **Table 1**. Any patients showed malignances in the last 5 years neither active infections at the moment that samples were taken. Peripheral blood mononuclear cells (PBMCs) were isolated by Ficoll-PaqueTM density gradient centrifugation and cryopreserved with 10% of DMSO in liquid nitrogen until their analysis.

**Table 1:** Clinical data of KTR groups and healthy controls.

	HC	TOL	CR	MO	TT
Number of patients	7	9	6	7	7
Donor (Living/deceased); n	–	5:4	1:5	4:3	2:5
Recipient age at enrolled; mean (range), y	51.2 (37-64)	50.7 (36-63)	48 (28-62)	50.4 (39-81)	40.5 (24-61)
Recipient age at transplant; mean (range), y	–	32.8 (21-58)	31.3 (7-48)	28.7 (14-48)	34.7 (18-55)
Recipient gender; n (M:F)	5:2	8:1	2:4	6:1	6:1
IS free; mean (range), y	–		–	–	–
Years IS free; mean (range),y	–	5.8 (1-9)	–	–	–
**HLA mismatches (mm); n**					
No mm	–	4	0	0	1
HLA (A or B) mm	–	0	1	0	0
HLA (A + B) mm	–	1	2	2	1
HLA (A + DR) mm	–	1	2	1	1
HLA (B + DR) mm	–	0	0	1	0
HLA (A, B, DR) mm	–	2	1	1	4
HLA (DR) mm	–	0	0	1	0
Missing data	–	1	0	1	0
**Donor-specific antibodies; n**					
No DSA	–	7	5	5	5
DSA class I	–	1	1	0	0
DSA class II	–	1	0	0	1
DSA class I + II	–	0	0	0	0
Missing data	–	0	0	2	1
**IS regimen; n**					
MMF	–	–	1	–	–
CNI + Steroids	–	–	1	–	–
MMF + Steroids	–	–	1	–	–
CNI + MMF	–	–	2	–	–
CNI + Aza + Steroids	–	–	1	–	–
Steroids	–	–	–	7	–
CNI + MMF + Steroids	–	–	–	–	7
**Renal function parameters; (mean ± SD)**					
Creatinine; nmols/L	–	98.5 ± 17.1	222.5 ± 133.6	91.4 ± 17.1	152.7± 55.3
eGFR; mL/min/1.73m^2^)	–	75.25 ± 19	33.6 ± 15.5	80.4 ± 12.4	51.4 ± 18.3
**Cell counts; (mean ± SD)**					
White blood cells x 10^9^	6.53 ± 1.5	6.75 ± 0.9	6.68 ± 1.7	6.58 ± 1.5	7.4 ± 0.9
Lymphocytes x 10^9^	2.2 ± 0.7	2.15 ± 0.5	0.95 ± 0.3	1.42 ± 0.3	1.78 ± 0.7

### DNA Extraction and Whole-Genome Methylation Profiling

DNA was extracted using an ATP Genomic DNA Mini Kit (ATP Biotech, Taipei, Taiwan) following the manufacturer’s instructions, then quantified with a Qubit 2.0 Fluorometer (Life Technologies, Carlsbad, CA, USA). Starting with 500 ng of high-quality genomic DNA, unmethylated cytosines were converted to uracils using an EZ DNA MethylationTM Kit (Zymo Research Corp. Irvine, CA, USA). Subsequently, whole-genome methylation profiles were characterized by amplification of converted DNA and their hybridization on InfiniumMethylationEPIC_v1.0 BeadChips Kits (Illumina Inc. San Diego, CA, USA) following Illumina’s Infinium HD Assay Methylation Protocol. Fluorescence intensities were measured with a HiScan apparatus (Illumina Inc. San Diego, CA, USA).

### Bisulfite Pyrosequencing

First, bisulfite modification was performed with 500 ng of total DNA using an EZ DNA methylation kit (Zymo Research). Modified DNA was amplified using pyrosequencing primers (**Supplementary Table 1**). DNA methylation levels were analyzed with the PyroMark kit (Qiagen, Hilden, Germany) and the PyroMark Q24 system (Biotage, Uppsala, Sweden), following the manufacturer’s protocol.

### DNA Methylation Analysis

Raw data for the analysis were extracted with Illumina’s Genome Studio data analysis software, in the form of a Genome Studio Final Report (sample probe profile). These data were analyzed within the R/Bioconductor statistical computing environment (www.r-project.org, www.bioconductor.org). Using the lumi Bioconductor package (https://bioconductor.org/packages/release/bioc/html/lumi.html), raw methylation data were background-corrected, log2-transformed, quantile-adjusted for color balance, and normalized. Probes not detected in at least one sample (p > 0.01) and sex chromosomes were excluded from subsequent analyses. Homogeneity of each defined sample-group was analyzed by principal component analysis (PCA). To detect differentially methylated probes, a linear model was fitted to the data and empirical Bayes-moderated t-statistics were calculated using the limma package from Bioconductor. Probabilities were adjusted by determining the false-discovery rates (FDR) using the Benjamin–Hochberg procedure. Probe sets with a differential M-value (log2 ratio of intensities of methylated probe *versus* unmethylated probe) of > 1.5 and an adjusted FDR p < 0.05 were considered to be differentially methylated. Gene ontology analysis of those genes associated with differentially methylated probes was performed with the DAVID web-based tool (https://david.ncifcrf.gov).

### Weighted Gene Co-Expression Network Analysis (WGCNA)

Co-methylation networks were constructed using the WGCNA package (11). Differential methylation analysis was performed as described above. For this analysis we selected probes with > 20% of variation in their average methylation value (β) and an adjusted-FDR p < 0.05 between any of the three clinical groups (HC, TOL and CR). Differential methylated regions with high absolute correlations within the network, and with a high topological overlap measure were clustered into modules. We then established a cut height of 1.1 to generate 18 correlated modules. Module eigengenes were defined as the first principal component of each gene module. Non-parametric Mann–Whitney tests were used to determine significant differences between the three groups. Functional interaction networks for each module were derived using STRING v10 (12). The resulting network was exported to Gephi (https://gephi.org) in which the Fruchterman–Reingold clustering algorithm was used to generate the final network.

### Cell-Type Deconvolution Analyses

MethylResolver was used to deconvolute bulk DNA methylation data into different cellular fractions (13). Specifically, the R package MethylResolver was used to deconvolute normalized beta values using the default leukocyte signature. The leukocyte signature comprises 419 optimal CpGs to deconvolute 11 leucocyte cell-types (Monocytes, Dendritic cells, Macrophages, Neutrophils, Eosinophils, Regulatory T cells, Naive T cells, Memory T cells, CD8 T cells, Natural Killer cells, and B cells). We also performed deconvolution using MethylCIBERSORT as implemented in MethylCIBERSORT R package (14). We used normalized beta values and Stromal_v2 signature. All deconvolutions showed highly significant values (P<0.01 and correlation > 0.9).

### Statistical Methods

Data in scatter dot plots are summarized as the median ± interquartile range. Non-parametric Mann–Whitney U tests were used to compare groups. Differences were considered to be statistically significant for values of p < 0.05. Statistical analyses were performed using Prism software, version 7 (Graph-Pad, La Jolla, CA) and IBM SPSS Sta-tistics for Windows, Version 20.0 (IBM Corp., Armonk, NY).

## Results

### Operational Tolerance and Chronic Rejection Are Associated With Distinct DNA Methylation Profiles

**Figure 1:**
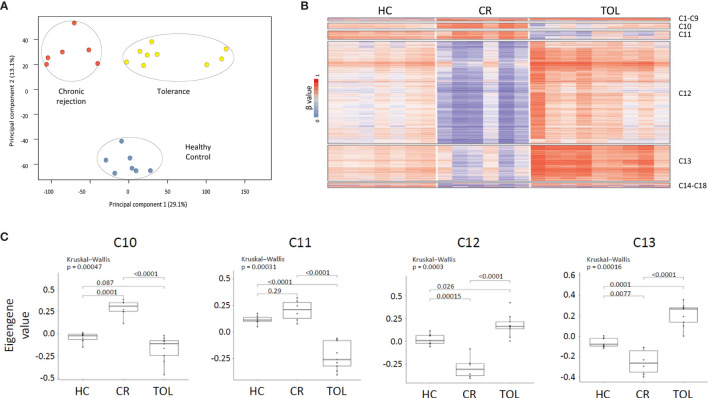
DNA methylation dynamics associated with transplant outcome. **(A)** PCA analysis of DNA methylation data from healthy controls (HC), chronic rejection (CR) and operational tolerance (TOL) kidney-transplanted recipients. **(B)** Weighted gene co-methylation network analysis in kidney transplant recipients. The analysis included all differentially methylated regions between HC, CR and TOL patients (β > 0.2, FDR <0.05). **(C)** Box plot of eigengene values in each gene cluster. These values are those of the first principal component of the DNA methylation data in each module. The significance of group differences was determined by the Kruskal-Wallis test, followed by a post-hoc test.

In order to study the methylation dynamics in peripheral blood associated with operational tolerance, we performed whole-genome DNA methylation analysis in PBMCs from KTR with operational tolerance (TOL; n = 9), chronic rejection (CR; n = 6), and healthy controls (HC; n = 7) (**Table 1**). In this method, we interrogated 850,000 genomic regions in each sample. Two-dimensional PCA showed a differential methylation profiles for all patient groups (**Figure 1A**), enabling us to identify 429 differentially methylated regions (DMRs) associated with 252 genes in the CR group and 524 DMRs (335 genes) in the TOL patients in comparison with healthy controls (**Supplementary Table 2**). Taking the transplantation event into account, the greatest differences at the DNA methylation level were observed between the CR and TOL groups, in which 6128 DMRs (2737 genes) were annotated, most which corresponded to hypomethylated CpG sites in CR (5662 DMRs), indicating that CR is associated with a hypomethylated profile in peripheral blood that it is not observed in healthy controls or operationally tolerant patients (**Supplementary Table 2**).

In spite of the low number of patients, two subgroups were clearly differentiated in TOL patients. PCA analysis revealed a set of three TOL patients (TOL-R) with the most DMRs (18,158) compared with CR patients (**Supplementary Figure 1**). By contrast, the other TOL patients (TOL-L; n=6) were closer to the CR group, with 2905 DMRs. Nonetheless, we did not observed methylation differences associated with time from transplantation and the clinical data available at this time gives us little clues of where the methylation differences between tolerant patients could be originated. In any case, gene ontology analysis showed that TOL-R was much more enriched in immune categories (**Supplementary Figure 1**), suggesting functional differences between both patient groups.

### Co-Methylation Network Analysis Reveals Distinct Epigenetic Profiles Associated With Operational Tolerance

To identify the epigenetic signatures associated with tolerance and chronic rejection we performed weighted gene co-expression network analysis (WGCNA). This method infers gene interconnections based on co-expression, which allows the generation of clusters of genes associated with the same pathways or functions, and correlates them with transplant status. We used this method to analyze DNA methylation data, so the resulting clusters represent epigenetic co-regulation, i.e., gene modules with similar methylation dynamics across all samples. First, we selected all probes that were differentially methylated (β > 0.2, FDR < 0.05) between any of the patients groups, which made 8078 DMRs available for analysis. This probe set generated 18 co-regulatory clusters after WGCNA, although most probes (> 85%) were concentrated in just four of these clusters (C10, C11, C12 and C13) (**Figure 1B** and **Supplementary Table 3**). As shown in the heatmap, most of the selected DMRs corresponded to hypomethylated CpG sites in the CR group, which were concentrated in clusters C12 (5223 DMRs) and C13 (1804 DMRs). Moreover, the methylation values across all samples showed that probes in C12 and C13 were hypomethylated in CR and hypermethylated in TOL patients relative to healthy volunteers (p < 0.05 between all sample groups) (**Figure 1C**). On the other hand, the C10 cluster showed the opposite trend, comprising those probes specifically hypomethylated in the TOL and hypermethylated in CR (p < 0.05) (**Figure 1C**). The C11 cluster showed similar results although the methylation differences between the CR patients and the healthy controls were not significantly different. Finally, genomic distribution of all the DMRs in the co-regulated network showed that they were preferentially associated with gene body regions rather than on promoters, and that they were mostly absent on CpG islands (**Supplementary Figure 2**).

**Figure 2:**
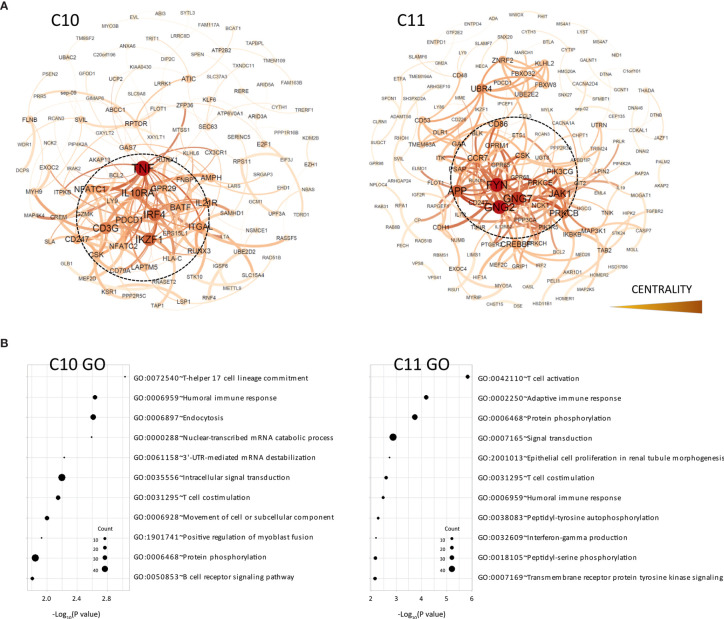
Functional analysis of clusters C10 and C11. **(A)** Functional interaction networks derived from co-methylated clusters (C10 and C11) obtained from WGCNA analysis, corresponding to DMRs hypomethylated in the TOL group. Network centrality is indicated by the color scale and node size. **(B)** Gene ontology analysis of clusters C10 and C11.

In order to evaluate whether these co-regulatory networks truly represent functional interaction within these modules, we generated protein–protein interaction networks from databases of physical interaction and databases of curated biological pathway knowledge using the STRING tool (**Figure 2A**). By this method, we observed that the cluster C10 and C11 was mostly comprised by protein interactions associated with immune functions. Thus, the tumor necrosis factor (TNF), a key factor of the lymphocyte differentiation and inflammation programs, was the most central gene in the C10 clusters. Similarly, the FYN proto-oncogene Src family tyrosine kinase (FYN), which is highly expressed in T cells and associated with TCR signaling, was the most interconnected gene within the network. Gene ontology analysis of the genes within the network showed high enrichment in immune functions, including T cell activation, humoral and adaptive immune response and Th17 lineage commitment (**Figure 2B** and **Supplementary Table 4**). We observed specific hypomethylation of some key genes in B cell development, including the ST6 beta-galactoside alpha-2,6-sialyltransferase 1 (ST6GAL1), a glycan-modifying enzyme involved in survival of transitional B cells, the membrane-spanning 4-domains A1 gene (MS4A1), which is a surface protein necessary for plasmatic cell differentiation, and the myocyte enhancer factor 2C (MEF2C), a transcription activator required for B cell activation and survival in response to BCR stimulation. We also observed a low level of methylation in some key genes of the Th17 differentiation program, such as the basic leucine zipper ATF-like transcription factor (BATF) and the interferon regulatory factor 4 (IRF4), both transcriptional regulators of RORC. The B and T lymphocyte attenuator (BTLA) and the programmed cell death protein 1 (PDCD1) inhibitory receptors, both CD28 family members, were also demethylated in TOL patients.

**Figure 3:**
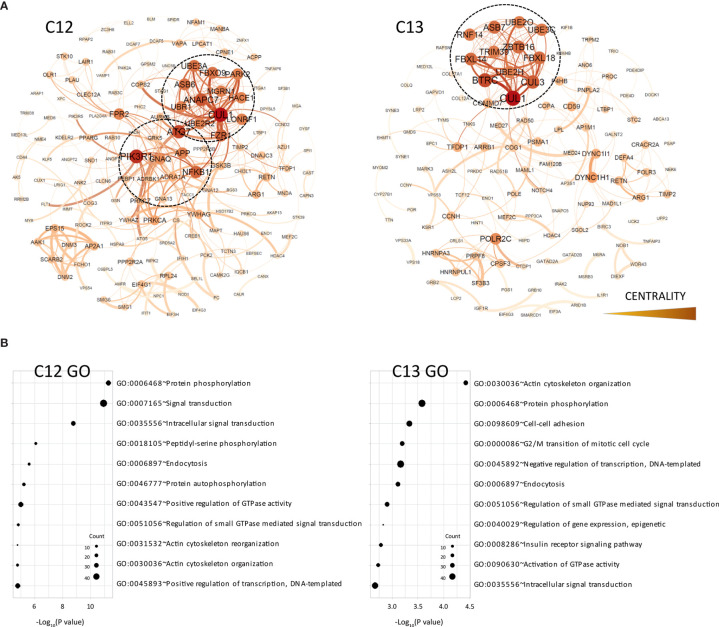
Functional analysis of clusters C12 and C13. **(A)** Functional interaction networks derived from co-methylated clusters (C12 and C3) obtained from WGCNA analysis, corresponding to DMRs hypomethylated in the CR group. **(B)** Gene ontology analysis of clusters C12 and C13.

On the other hand, the C12 and C13 clusters, which included most genes hypomethylated in CR patients, showed very different functional networks which were organized around genes associated with ubiquitination pathways (UBR1, ANAPC7, UBE3A, UB3C, etc.) (**Figure 3A**). In both clusters, DMRs associated with the cullin 1 gene (CUL1) occupied the most central position within the functional network. This gene is a core component of the E3 ubiquitin-protein ligase complex, mostly expressed by T and B lymphocytes in peripheral blood and associated with cellular activation (15). These clusters also showed a very different pattern of functional enrichment, mostly associated with cellular processes such as endocytosis, signal transduction, protein phosphorylation and cell adhesion (**Figure 3B**). However, we did observe demethylation in some genes associated with antigen presentation (CALR), B cell functions (LYN) and other genes involved in B cell development, such as PRKCB and NFAM1.

**Figure 4:**
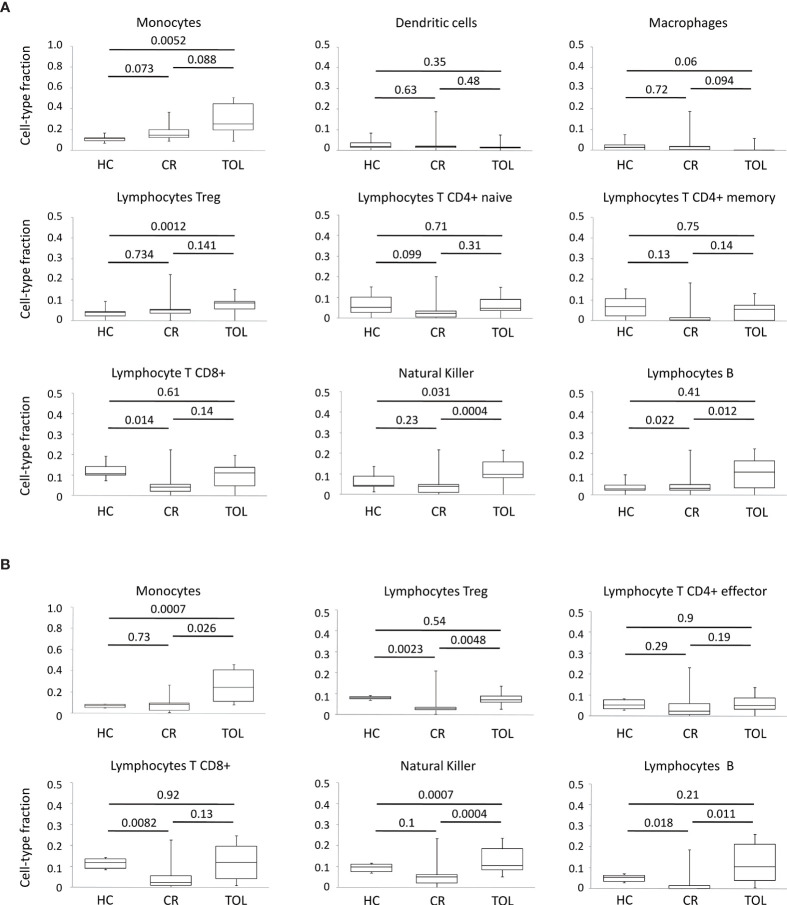
Deconvolution analysis of DNA methylation data in KTR. **(A)** Boxplots for cellular deconvolution by MethylResolver. **(B)** Boxplots for cellular deconvolution by MethylCIBERSORT. P-values for Wilcoxon’s Rank Sum Tests are shown.

In general, these results indicated that the genes in the major clusters derived from our co-regulatory networks are mostly associated with the epigenetic modulation of lymphocyte differentiation and co-stimulation pathways in tolerance, and with intracellular signaling and ubiquitination mechanisms in chronic rejection. Nonetheless, is important to note that these DNA methylation profiles are likely to be influenced by changes in the cellular composition of the samples. In order to study this possibility, we perform *in-silico* immune cell deconvolution of methylation profiles in all samples using two different methods: MethylResolver and MethylCIBERSORT (**Figure 4**). With both methods, we observed that TOL patients showed higher levels of monocytes, NK cells and B lymphocytes, suggesting that the DNA methylation signature in tolerance is at least partially associated with changes in these populations.

### Stable Patients With Low Doses of Prednisone Show Methylation Patterns Close to Tolerance

**Figure 5:**
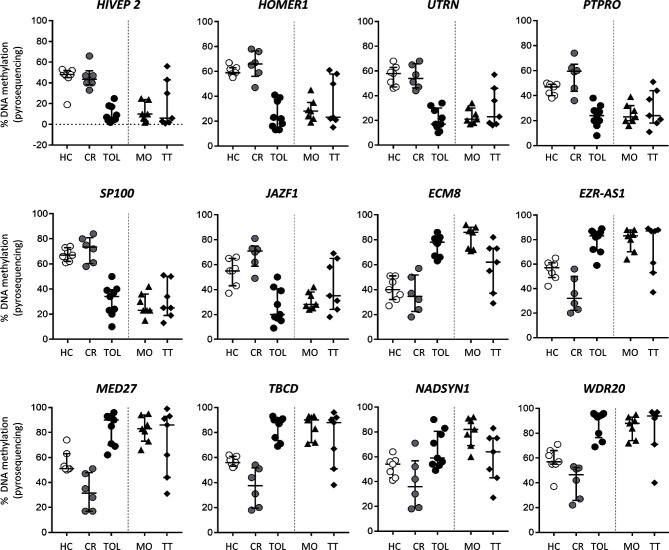
DNA methylation analysis of kidney transplant recipients under different immunosuppressive treatment. Bisulfite pyrosequencing of selected DMRs in operationally tolerant patients (TOL), patients with low-dose glucocorticoids as monotherapy (MO) and patients with standard triple-therapy (TT). Results are shown for each patient; lines show the median ± interquartile range.

Tolerant and CR patient groups represent opposite extremes of kidney transplant outcomes, so we wanted to evaluate whether these methylation signatures were correlated with the intermediate outcomes observed in stable patients receiving monotherapy (low doses of prednisone) or who were on standard triple therapy. With this aim, we used weighted gene co-methylation network analysis to select a set of representative DMRs for each patient group. First, we identified in each co-methylation cluster the hub DMRs that may orchestrate module behavior, defined by high module membership (representing connectivity within the co-methylation network) and strong correlation with the clinical features (TOL and CR). These parameters showed a linear correlation in the four major clusters derived from the network (C10-C13) (**Supplementary Figure 3**). Hub DMRs were identified as the top 10% of ranked DMRs with the highest membership value and an FDR < 10-4. From this list, we selected DMRs in the C10 and C13 clusters, since these showed the greatest differences between the CR and TOL groups. Using this criterion, we analyzed six candidates in the C10 cluster (associated with the genes HIVEP2, HOMER1, UTRN, PTPRO, SP100 and JAZF1), and in cluster C13 (associated with EMZ8, EZR-AS1, WDR20, NADSYN1, TBCD and MED17). In addition, these DMRs were selected because their methylation values were very consistent across all samples. First, DNA methylation values in this set of DMRs were validated by bisulfite pyrosequencing in the same sample patients as those used for microarray analysis to confirm the results of the array analysis by a different method. Results from pyrosequencing analysis confirmed the significant differences between CR and TOL groups for all genes (**Supplementary Figure 4**). DMRs derived from the C10 cluster showed very low levels of methylation in TOL compared with CR and healthy controls and, conversely, C13 probes showed very high levels of methylation in TOL patients relative to the other two groups. Additionally, we evaluated the DNA methylation of these DMRs in a new cohort of stable KTR under monotherapy with a low dose of glucocorticoids (MO; n = 7), or standard triple-therapy based (TT group; n = 7). DNA methylation in the MO group showed very similar patterns to those of patients with TOL (**Figure 5**). However, the DNA methylation levels of stable patients from the TT group were more diverse, some patients were close to the TOL group, but others showed DNA methylation patterns more similar to those of the CR group, which may reflect a different immunological status among those stable patients (**Figure 5**). Despite the small number of samples, we can determine that clinically stable patients with reduced IS (MO group) had a DNA methylation pattern near to tolerance, whilst stable patients under standard IS were more variable.

## Discussion

Transcriptomic studies and immunophenotyping analysis in peripheral blood have been used to derive tolerance signatures in KTR, although the epigenetic networks regulating these transcriptional signatures have not yet been studied. Here, we demonstrated that genome-wide DNA methylation analysis can provide reliable epigenetic signatures associated with chronic rejection and operational tolerance in KTR. However, two fundamental questions arising are how omic approaches (transcriptomic and/or epigenomic) reflect the immune status of the recipient and whether they can be used to develop predictable signatures for operational tolerance that may help to reduce immunosuppressive treatment.

Previous transcriptomic analyses have shown that transcriptional signatures in TOL patients are associated with an enriched B cell profile (4–6). This result is clearly consistent with those of some flow cytometry studies, which have shown higher frequencies of circulating of B cells in those patients, specifically of the naive and transitional populations (16). The role of these cells in operational tolerance is not fully understood, although it may be related to some regulatory functions that can be exerted on effector T cells (17) or the ability of transitional B cells to produce IL10 and to inhibit CD4 T cell responses (18, 19) Consistent with these results, we observed DNA demethylation in TOL patients of genes associated with the B cell program. Some of these genes, such as ST6GAL1 and MS4A1 (encoding CD20), have been specifically associated with the survival of transitional B cells and B cell function (20, 21) and, consequently, demethylation in peripheral blood of TOL patients suggests a specific expansion of these populations.

A meta-analysis of 96 tolerant samples from five studies has reported a common transcriptomic signature to be expressed in peripheral blood that is centered on B and T cell proliferation genes and the inhibition of CD14 monocyte-related functions (22) We have confirmed that DNA methylation analysis also reflects changes in B and T cell populations in blood although, in general, we observed only a moderate correlation between epigenetic changes and the transcriptomic signature derived from this meta-analysis. In fact, we only found five differentially methylated genes associated with operational tolerance (BLK, IRF4, ID3, HINT1 and PLB1) among the top-20 genes reported in the transcription meta-signature. This result indicates that epigenomic and transcriptomic signatures are not necessarily equivalent and perhaps reflect the different molecular and cellular traits associated with operational tolerance. On the other hand, it is very important to emphasize that a recent study has demonstrated that the transitional B cell signature associated with tolerance may be partially induced by the immuno-suppressive treatment (23). This led to a new transcriptional signature without this treatment bias being derived. This new signature initially identified 28 differentially expressed genes, and only eight of them (RAB40C, TNFAIP3, IRF2, PDE4B, DNMT3A, SEC24D, HP and ITGB1BP1) showed differential DNA methylation in our analysis, a result expected because we did not analyze the epigenome of patients with different immunosuppressive treatments.

In addition to B cell function, poor Th17 response has been associated with operational tolerance and prolonged graft survival, whereas CR patients had a higher frequency of Th17 cells and greater TCR signaling (24, 25). We observed differential methylation of genes associated with the activation and costimulation of T-cells, and the Th17 differentiation program between TOL and CR patients. It is interesting to highlight that several transcriptional regulators of RORγ (LY9, BATF and IRF4), which is the master regulator of Th17 differentiation, were hypomethylated in the tolerant state. Given that loss of methylation in gene promoter regions is usually associated with greater transcriptional potential, our results do not suggest that there is a smaller Th17 population in peripheral blood. In any case, we cannot exclude that other molecular pathways associated with Th17 functions may be altered in TOL patients. In fact, we observe de-methylation of the BTLA and PD1 costimulatory molecules and genes associated with the negative regulation of ERK and NFkB pathways, whose expression might damage the T-cell activation and Th17 response, as it has been previously reported (25, 26).

Another interesting finding in our study was the clear hypomethylation signature associated with the CR state. In fact, we found 5662 DMRs that were specifically demethylated in CR patients compared with tolerant patients, but only 466 that were demethylated in TOL compared with CR patients. It is not clear what this obvious bias towards loss of methylation in CR implies, but we and other researchers have demonstrated that immune cell activation and differentiation are associated with a genome-wide demethylation wave in many immune-related genes (27–30). Some of the genes associated with immune function were demethylated, although GO analysis showed preferential enrichment in genes involved in cell signaling pathways. Analysis of the functional interactions between the annotated DMRs showed that the loss of methylation was closely associated with ubiquitination pathways, which are involved in protein degradation, antigen presentation, TCR and BCR signaling, and innate immunity (31). Some E3 ubi-quitin protein ligases have been associated with peripheral tolerance (32–34) and inhibitors of the ubiquitin-proteasome system have been tested in order to improve cold organ preservation, especially for liver transplant (35). However, the role of these pathways in transplant rejection remains to be determined.

On the other hand, we did select the DMRs that were most representative of the operational tolerance state and analyzed them in stable patients receiving treatment with a low dose of glucocorticoids as their only therapy, or with standard triple-therapy. Of the battery of tested genes, some were mechanistically associated with immune response and tolerance although they have not yet been specifically studied in organ transplantation. Thus, JAZF1 is a negative regulator of IFN-γ and IL-17 in macrophages (36), SP110 modulates nuclear factor-κB (NF-κB) activity (37), and the PTPRO encodes a receptor-type tyrosine kinase essential for B cell receptor signaling and associated with acute rejection in a genome-wide association study (38). Notably, DNA methylation levels in the analyzed genes were nearly identical in recipients under monotherapy and in TOL patients, suggesting that the DNA methylation pattern could reproduce the observed good clinical outcome of the graft. We cannot rule out the possibility that DNA methylation can be also be biased by a cofounding effect due to immunosuppressive therapy. Further studies with KTRs groups under various immunosuppressant regimens will be necessary to dissect the specific contribution of immunosuppression. Nonetheless, DNA methylation profiles between healthy controls and “tolerant” patients, both free from IS, are significantly different, suggesting that DNA methylation changes reflect a favorable immune response in tolerant patients rather than the beneficial effect of drug withdrawal. In any case, this effect will have to be accounted for in future epigenetic studies determining the methylation changes throughout transplant evolution and in stable patients with different immunosuppressant regimens.

## Conclusions

Although only a limited number of patients have been included in this study, our results demonstrate that epigenetic dynamics in mononuclear cells from peripheral blood are associated with kidney transplant outcome. Tolerant patients develop a specific DNA methylation pattern, providing proof of concept for the feasibility of using methylation analysis to monitor stable patients with good outcome. Further studies with larger cohorts and patients receiving various immunosuppressive regimens will allow us to develop reliable epigenetic biomarkers that will help reduce the immunosuppressant therapy, in combination with clinical criteria in patients with methylation profiles closer to tolerance.

## Data Availability Statement

The datasets presented in this study can be found in online repositories. The names of the repository/repositories and accession number(s) can be found below: (https://www.ncbi.nlm.nih.gov/geo/), GSE156359.

## Ethics Statement

The studies involving human participants were reviewed and approved by Institute of Child Health/Great Ormond Street Hospital (Research Ethics Reference: 09/H0713/12). The patients/participants provided their written informed consent to participate in this study.

## Author Contributions

RR, MH-F, BS-A and CL-L designed the research, analyzed the data, and supervised the manuscript. RR wrote the paper. AA, JL, AC and IM analyzed data. RR, VC-I, MS, MS-F, EC, AL-V and CD-C did the research. All authors contributed to the article and approved the submitted version.

## Funding

This research was funded by the Plan Nacional de I+D+I 2013-2016 ISCIII (Spanish Institute of Health Carlos III, grant numbers PI16/01318, PI17/01244, and PI19/00184), Gobierno del Principado de Asturias, PCTI-Plan de Ciencia, Tecnología e Innovación 2018-2022 (Grant number IDI/2018/144), FEDER Funding Program of the European Union, the Agencia Estatal de Investiga-cion (AEI) (Ayuda Juan de la Cierva-Incorporación, IJCI-2017-33347 to RR), the Red Española de Investigación Renal (REDinREN, grant number RD16/0009/0020), Severo Ochoa Excellence accreditation (SEV-2016-0644) and the Basque Department of Industry, Tourism and Trade (Etortek and Elkartek programs). The GAMBIT study acknowledges financial support from FP7-HEALTH-2012-INNOVATION-1 (project number 305147: BIO-DrIM) and project HEALTH-F5–2010–260687, The ONE Study. Medical Research Council MRC grants to MH-F [G0801537/ID:88245] and to MRC Centre for Transplantation [MRC grant no. MR/J006742/1]. The research was funded/supported by the National Institute for Health Research (NIHR) Biomedical Research Centre based at Guy’s and St Thomas’ NHS Foundation Trust and King’s College London. All UK based centres received service support through Clinical Research Networks [study portfolio number 7521].

## Author Disclaimer

The views expressed are those of the author(s) and not necessarily those of the NHS, the NIHR, or the Department of Health.

## Conflict of Interest

Author MH-F is employed by UCB Celltech.

The remaining authors declare that the research was conducted in the absence of any commercial or financial relationships that could be construed as a potential conflict of interest.

## Publisher’s Note

All claims expressed in this article are solely those of the authors and do not necessarily represent those of their affiliated organizations, or those of the publisher, the editors and the reviewers. Any product that may be evaluated in this article, or claim that may be made by its manufacturer, is not guaranteed or endorsed by the publisher.
