# Supplementary material for: Defining a Methylation Signature Associated With Operational Tolerance in Kidney Transplant Recipients
Source: Front Immunol. 2021 Aug 20;12:709164. doi: 10.3389/fimmu.2021.709164 (PMC8417883; doi:10.3389/fimmu.2021.709164)
Supplement: Supplementary file 1 [file DataSheet_1.pdf]

Supplementary Material

Supplementary Figure 1. DNA methylation in chronic rejection and different tolerant patient subgroups.

Supplementary Figure 2. Distribution of DMRs according to CpG content and gene location.

Supplementary Figure 3. Identification of Hub DMR by WGCNA.

Supplementary Figure 4. Validation of microarray data by bisulfite pyrosequencing.

Supplementary Table 1. Pyrosequencing primers. Biotinylated primers are indicated in bold.

Supplementary Table 2. Differentially methylated regions (DMRs) between HC, CR and TOL groups.

Supplementary Table 3. DMR modules obtained by weighted gene co-methylation network analysis.

Supplementary Table 4. Gene ontology analysis of the co-regulated genes in each of the main modules (C10-C13) derived from WGCNA.

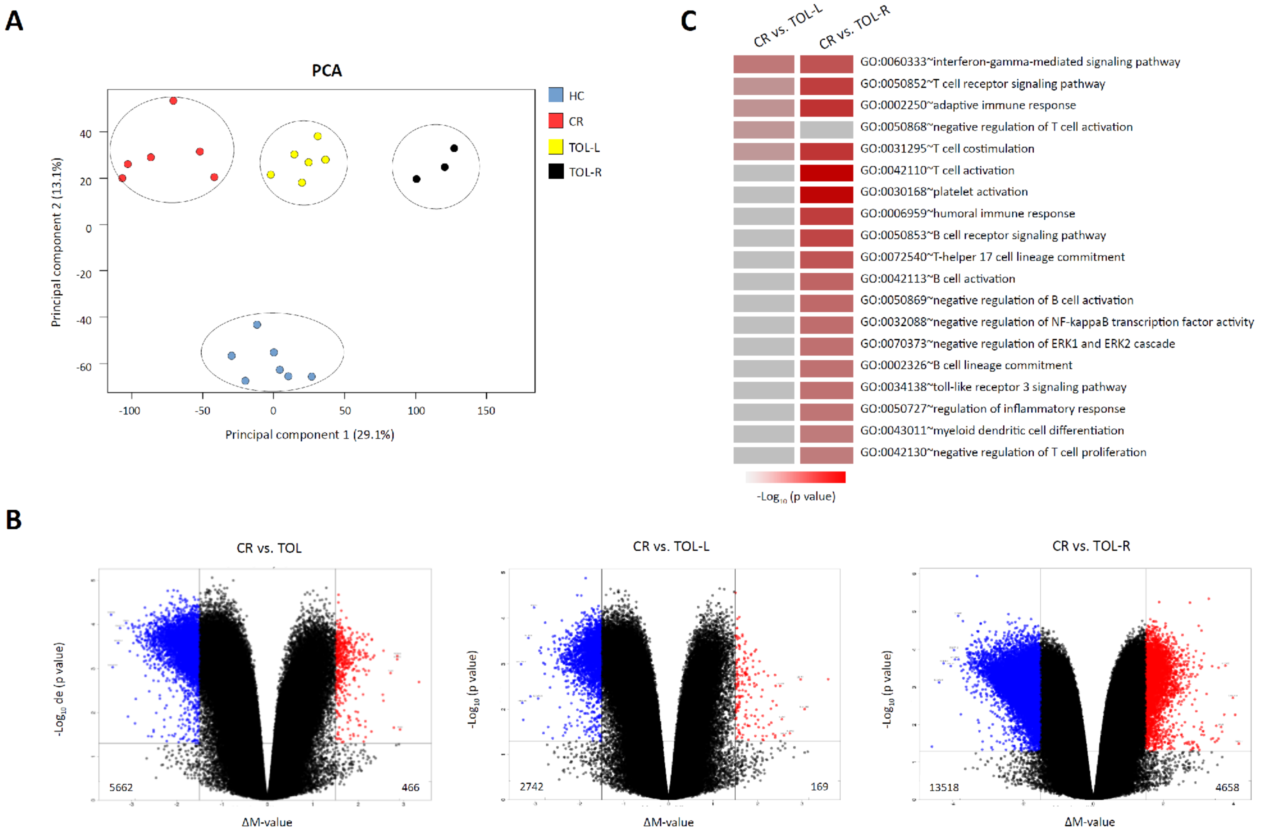

Supplementary Figure 1. DNA methylation in chronic rejection and different tolerant patient subgroups. A) PCA analysis of DNA methylation data. B) Volcano plots showing differentially methylated regions in CR vs. all TOL, CR vs. TOL-L and CR vs. TOL-R patients. Hypomethylated DMRs in the CR and TOL are shown in blue and red, respectively. C) Heatmap of the most significant GO terms associated with immune function in CR vs. TOL-L and CR vs. TOL-R.

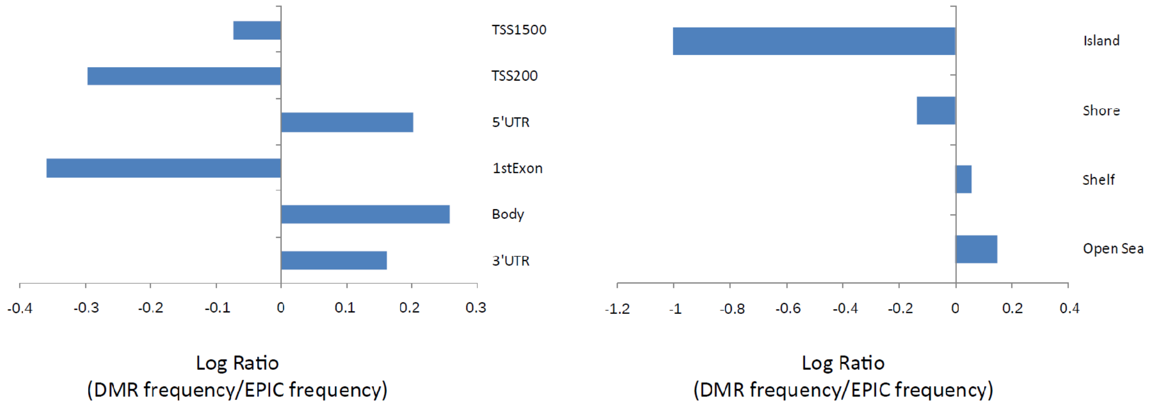

Supplementary Figure 2. Distribution of DMRs according to CpG content and gene location. Results are represented as the ratio between observed and expected frequencies.

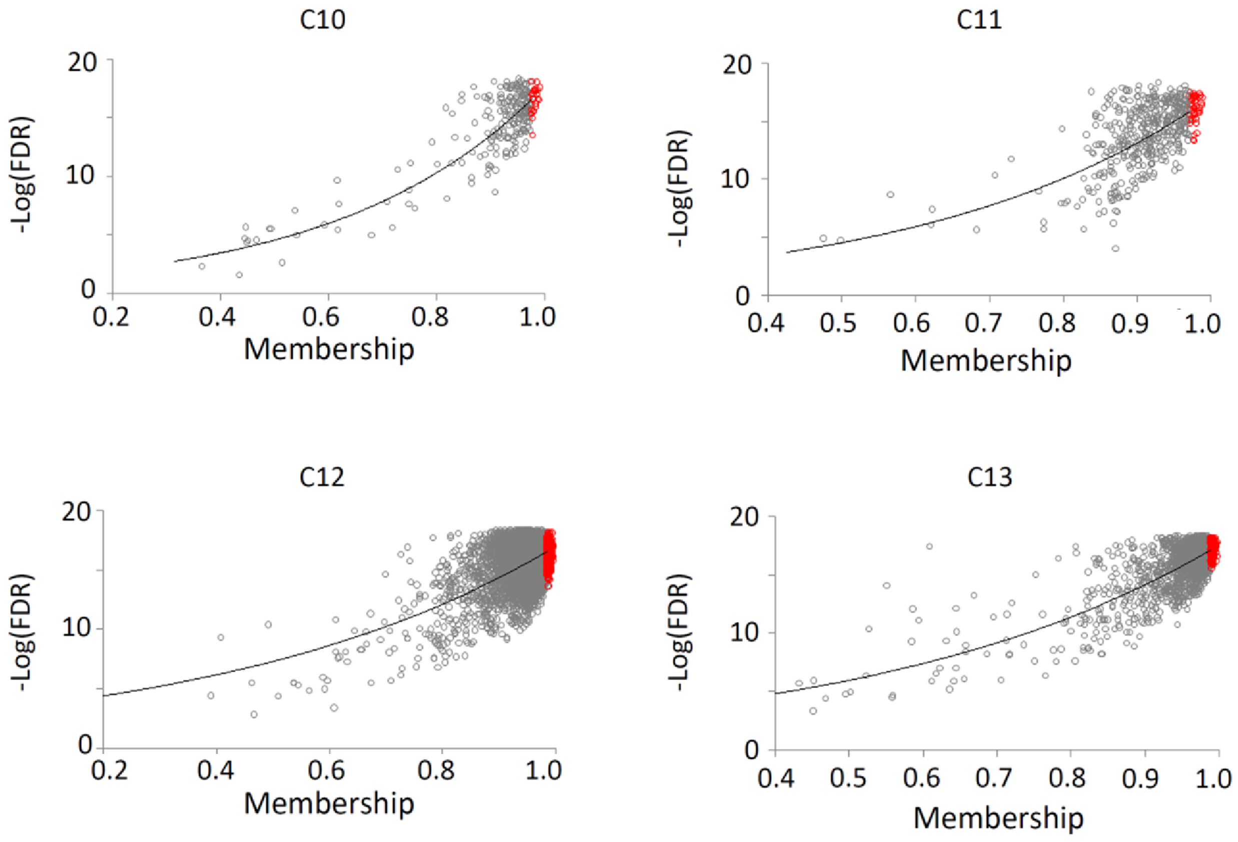

Supplementary Figure 3. Identification of Hub DMR by WGCNA. Scatter plot of module membership (a measure of network connectivity) and the correlation with clinical features (operational tolerance and chronic rejection).

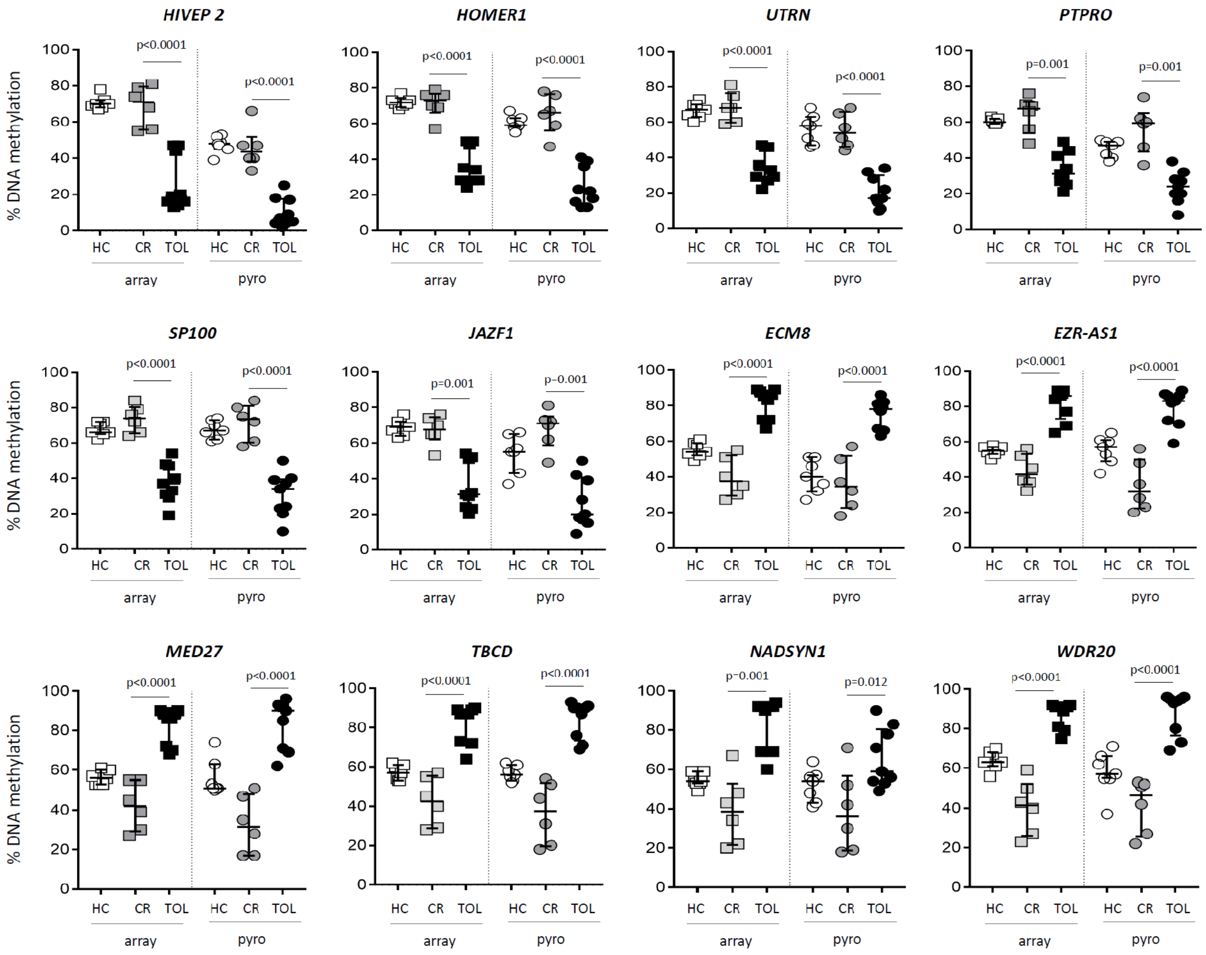

Supplementary Figure 4. Validation of microarray data by bisulfite pyrosequencing. DNA methylation results obtained by microarray analysis were validated in 12 selected DMRs by bisulfite pyrosequencing. Data are shown for each patient, line shows the median ± interquartile range. The significance of group differences was determined by the Mann–Whitney test. HC; healthy controls, CR; chronic rejection, TOL; tolerant patients.
